# Habitat constraints and self-thinning shape Mediterranean red coral deep population structure: implications for conservation practice

**DOI:** 10.1038/srep23322

**Published:** 2016-03-18

**Authors:** Alessandro Cau, Lorenzo Bramanti, Rita Cannas, Maria Cristina Follesa, Michela Angiolillo, Simonepietro Canese, Marzia Bo, Danila Cuccu, Katell Guizien

**Affiliations:** 1 Department of Architecture, Design and Urban planning, University of Sassari, Alghero, Italy; 2 Department of Life and Environmental Sciences, University of Cagliari, Cagliari, Italy; 3 Sorbonne Universités, UPMC Univ Paris 06, CNRS, Laboratoire d’Ecogéochimie des Environnements Benthiques (LECOB), Observatoire Océanologique, F-66650, Banyuls sur Mer, France; 4 Department III ‘Tutela degli Habitat e della Biodiversità Marina’, CRA 15 – ISPRA, Roma, Italy; 5 Dipartimento per lo Studio del Territorio e delle sue Risorse, Università degli Studi di Genova, Genova, Italy

## Abstract

The Mediterranean red coral, *Corallium rubrum*, is one of the most precious corals worldwide. Below 50 m depth, *C. rubrum* populations are generally characterised by large and sparse colonies, whereas shallow populations (above 50 m depth) show high densities of small colonies. We show here instead that populations dwelling between 80 and 170 m depth exhibited a continuous range of population density (from 2 to 75 colonies per 0.25 m^2^), with less than 1% of variance explained by water depth. An inverse relationship between maximum population density and mean colony height was found, suggesting that self-thinning processes may shape population structure. Moreover, demographically young populations composed of small and dense colonies dominated along rocky vertical walls, whereas mature populations characterised by large and sparsely distributed colonies were found only in horizontal beds not covered by sediment. We hypothesise that, in the long term, shallow protected populations should resemble to present deep populations, with sparsely distributed large colonies. Since the density of red coral colonies can decay as a result of self-thinning mechanisms, we advise that future protection strategies should be based also on a measure of red coral spatial coverage instead of population density.

Sessile organisms such as hard branching corals enhance the overall complexity of benthic ecosystems due to their three-dimensional erect structure, playing a prominent role in the pelagic–benthic transfer of energy^1^,^2^. Only few of them, however, are regarded as precious items targeted by professional fisheries^3^,^4^.

The Mediterranean red coral *Corallium rubrum* (Linnaeus, 1758) is a long-lived, slow-growing species, distributed throughout the Mediterranean Sea and adjacent Atlantic coasts, where it can be found between 10 and 800 m depth^5^, but more commonly between 30 and 200 m^6^,^7^. This species has gained considerable attention from the scientific community because of its cultural and economic relevance, which is linked to the use of its red calcium carbonate skeleton in the jewelry market worldwide^3^.

Harvesting practices with non-selective gears (*i.e.*, *ingenium* or St. Andrew cross) exist since ancient times (historical records of fishing practice with these gears date back to the fourth century B.C.)^3^,^7^,^8^, and since the late 1950s, the development of scuba diving technology has enabled additional selective harvesting in shallow-water populations (shallower than 50 m depth), allowing fishermen to (ideally) target only large and thus fecund colonies (*i.e.*, producing a high quantity of larvae)^6^,^9^,^10^. Catches of *Corallium rubrum* in the Mediterranean basin dramatically dropped between 1976 and 1984^7^, raising concern that populations were becoming over-exploited^11^. Demographic studies in shallow environments showed population structures skewed towards small-sized colonies^12^,^13^, confirming the risk of local extirpation and raising concern on the need of conservation and management^3^,^14^.

Until recently, *C. rubrum* harvesting has been regulated only at the national level, often in separate and uncoordinated ways in the different Mediterranean countries. Only in 2011 and 2012, FAO-GFCM (General Fishery Commission for the Mediterranean) adopted binding recommendations for the member states, imposing minimum size and depth limits (Recommendation GFCM/35/2011/2 and GFCM/36/2012/1). Nonetheless, in Sardinia (the area of the present investigation), a comprehensive management plan is already in place since 1989. The plan imposes additional and stricter regulations than those imposed by the GFCM, with a minimum catch size of 10 mm of basal diameter (7 mm for GFCM), and a depth limit of 80 m depth (50 m for GFCM)^7^,^15^.

Given the progressive impoverishment of shallow populations and the recently applied protection measures, professional fisheries have progressively shifted deeper, extending the risk of overexploitation to deep-dwelling populations (deeper than 50 m). As a consequence, an adaptive management plan is being developed by the FAO/GFCM^14^,^15^.

At present, the distribution of deep-dwelling populations of *C. rubrum* is still poorly known. The recent development of submarine technologies such as Remotely Operated Vehicles (ROVs) has allowed scientists to extend investigations to previously out-of-reach sites and depths via *non-invasive* direct observations that are particularly suited for endangered and/or overexploited species^16^. The few ROV-based investigations performed on deep-dwelling populations across the Mediterranean basin^4^,^6^,^10^,^17^,^18^,^19^ recorded mostly large and highly branched colonies, distributed sparsely or forming small patches on rocky surfaces^20^. From these observations, the hypothesis that *C. rubrum* population structure follows a depth-related distribution, with small and densely packed colonies in shallow environments and large and sparsely distributed colonies in deep ones was formulated^6^. Strikingly, this hypothesis opposes the classical view that ecosystem productivity decreases with increasing water depth: shallow areas receive both light energy and nutrient inputs, leading to a high primary production and supporting high secondary production^21^. Thus, in shallow environments it should be expected to find larger red coral colonies than in deep environments; the opposite pattern observed has been explained by more frequent disturbances in the shallow environment, either of anthropogenic^7^,^22^ or environmental origin^23^,^24^,^25^,^26^.

Under this assumption, demographic models predict that disturbances (either natural or anthropogenic) that cause the removal of large colonies should maintain the coral forest (*sensu* Rossi^27^) in a transient colonization stage (disturbed population) characterised by a high density of small colonies, or even lead to local extirpation when the different disturbances act concurrently^28^. Stable populations should then be characterized by large, highly branched and sparsely distributed colonies, structures that are often found in deep dwelling populations^6^. However, demographic models used to project red coral population structure under different pressures also included the assumption that the *C. rubrum* population are regulated by a density-dependent recruitment limitation. This process leads to population self-thinning: an intra-specific competition process in which space limitations link population density to the mean individual ground cover^29^.

In the case of *C. rubrum*, the role of intra-specific competition in regulating distribution and population structure of populations approaching a saturating density is still unexplored^30^,^31^. The deep environment offers the opportunity to investigate self-thinning assumptions, as, differently to what happened in shallow environments, the structure of the populations has not yet been completely modified by the intense selective harvesting pressure on large colonies. Even in the absence of intra-specific regulation on population demography, the distribution of benthic organisms can be influenced by habitat heterogeneity and complexity^32^,^33^. Habitat suitability also regulates larval settlement and post-settlement survival, through mechanisms involving different habitat characteristics (slope, roughness, sediment presence/absence, hydrodynamic intensity^34^).

Taking advantage of new technological tools, which provide data on population density and mean colony size in deep-dwelling populations of *C. rubrum*, this study aims to test three null hypotheses: (1) population density is not related to depth (2) population structure in the deep environment does not depend on habitat quality, namely, slope and sediment accumulation and (3) population density is not related to mean colony size.

## Results

**Figure 1:**
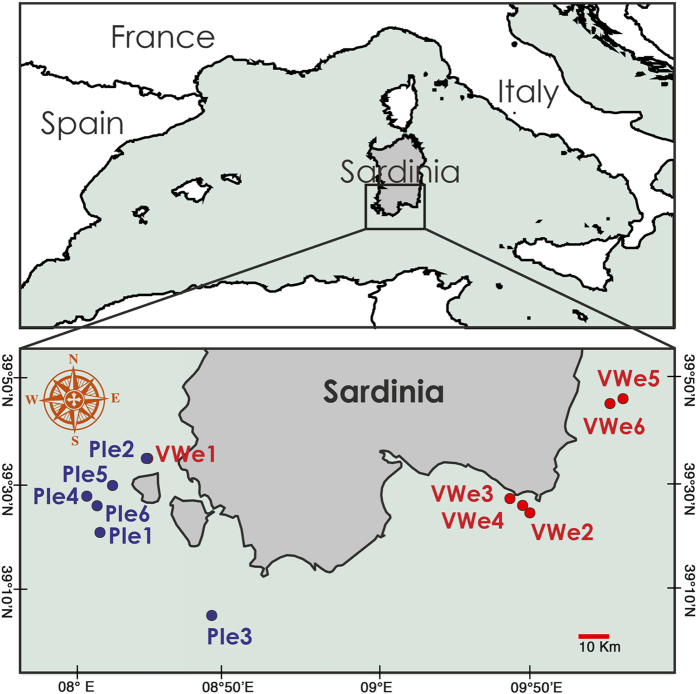
Map of the study area. Red and blue dots show the sites defined as ‘vertical wall environments’ (VWe), and ‘pinnacle environments’ (PIe), respectively. The map was generated using R software (package ‘rworldmap’, ‘getmap’ function) and modified in Adobe Photoshop, ‘CC 2014’ version.

**Figure 2:**
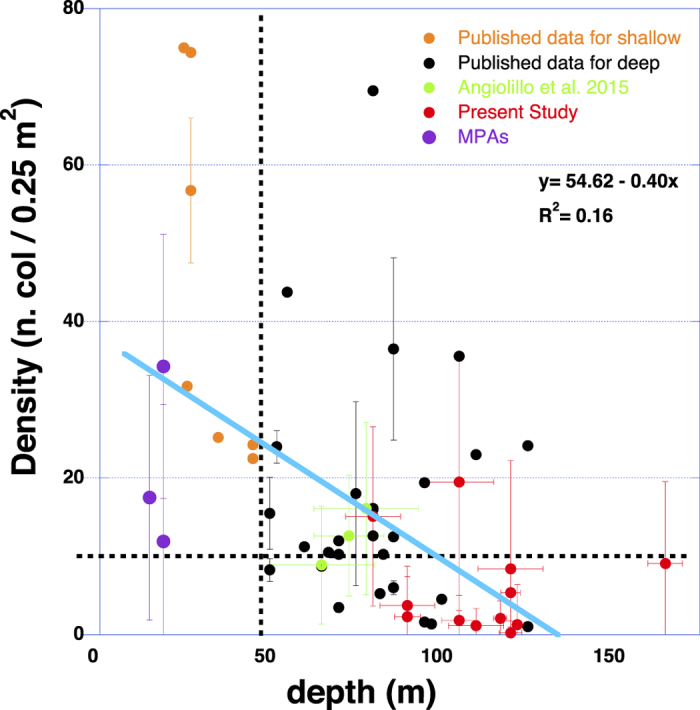
Scatter plot of density (number of colonies per 0.25 m^2^ ± S.D.) in relation to depth (m ± S.D.) from present study and published data on shallow and deep dwelling populations of *C.* rubrum. Standard deviation is reported only for data where the information was available. The data summarize 53 sampling sites from the Mediterranean basin (including coasts from Spain, France, Italy and results from the present investigation). The low explanatory power of depth (*R*^*2*^ = 0.16) suggests the absence of a bathymetric pattern.

Overall, a total of 3,676 colonies of *C. rubrum* were counted and measured, with a probability of occurrence of 49.2% within the 600 sampling units (*i.e.*, 0.5 × 0.5 m squares). Population density ranged from 1 to 75 colonies 0.25 m^−2^ over the 80–170 m bathymetric range (Figs 1,2, Supplementary Table S1).

Water depth explains less than 1% of the observed variance in red coral population density in the present study (R^2^ = 0.0062, P<0.001, water depth ranging from 80 to 170 m). The inclusion of data on deep red coral populations from literature (all data deeper than 50 m) only increased the explanatory power of depth to 2%. When the analysis was further extended to the full bathymetric range (20–180 m depth), including data from literature on shallow populations (shallower than 50 m) the explanatory power of water depth increased to 15% (R^2^ = 0.15; P < 0.001; Fig. 2). It is noteworthy that while in deep dwelling populations (deeper than 50 m), both high densities (>10 colonies) and low densities (<10 colonies) can be observed (Fig. 2), in shallow water populations only high colony densities (higher than 10 colonies 0.25 m^−2^) can be found, with the lowest densities being recorded in the oldest Marine Protected Areas (MPAs; Fig. 2).

**Figure 3:**
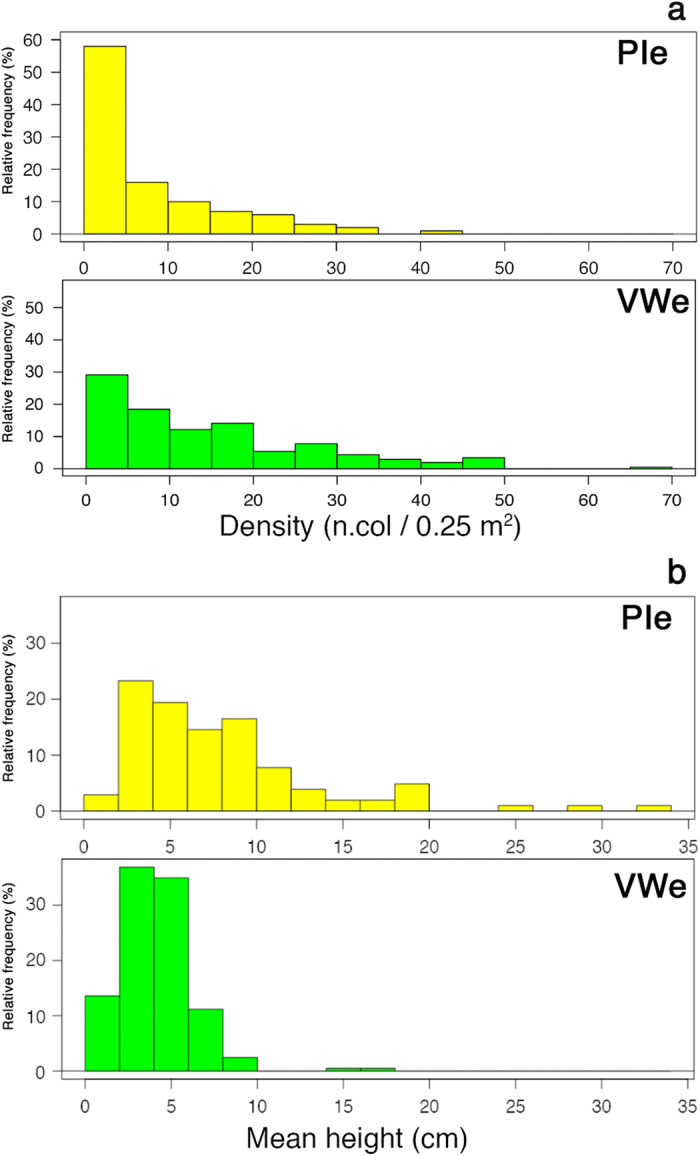
(**a**) Density and (**b**) size structure of *C. rubrum* populations from the two investigated substrates: PIe (yellow colored) and VWe (green colored); n = 600 and n = 319 for density and size, respectively.

Focusing on the deep environment, population densities estimated at meso-scale (tens/hundreds of kilometers) varied significantly between “vertical-wall” condition (VWe, transects performed on sites with average slope steeper than 40°) and pinnacle (PIe, average slope lower than 40°) condition (Mann-Whitney, *U* = 3.19^4^; *P* = 2.34^−11^), with values in VWe higher than in PIe (8.59 ± 0.71 and 2.93 ± 0.16 colonies 0.25 m^−2^ respectively; mean ± SE; Fig. 3). The population structure also differed between the two environments (Mann-Whitney, *U* = 5695; P < 0.0001), with colonies in PIe larger than in VWe (8.04 ± 0.57 cm and 4.37 ± 0.29 cm for PIe and VWe respectively; mean colony height ± SE). Such differences in population structure translate into significantly less-skewed size distributions in VWe than in PIe (Skewness = 0.787 in VWe and 1.98 in PIe; *P* < 0.0001; Fig. 3).

**Figure 4:**
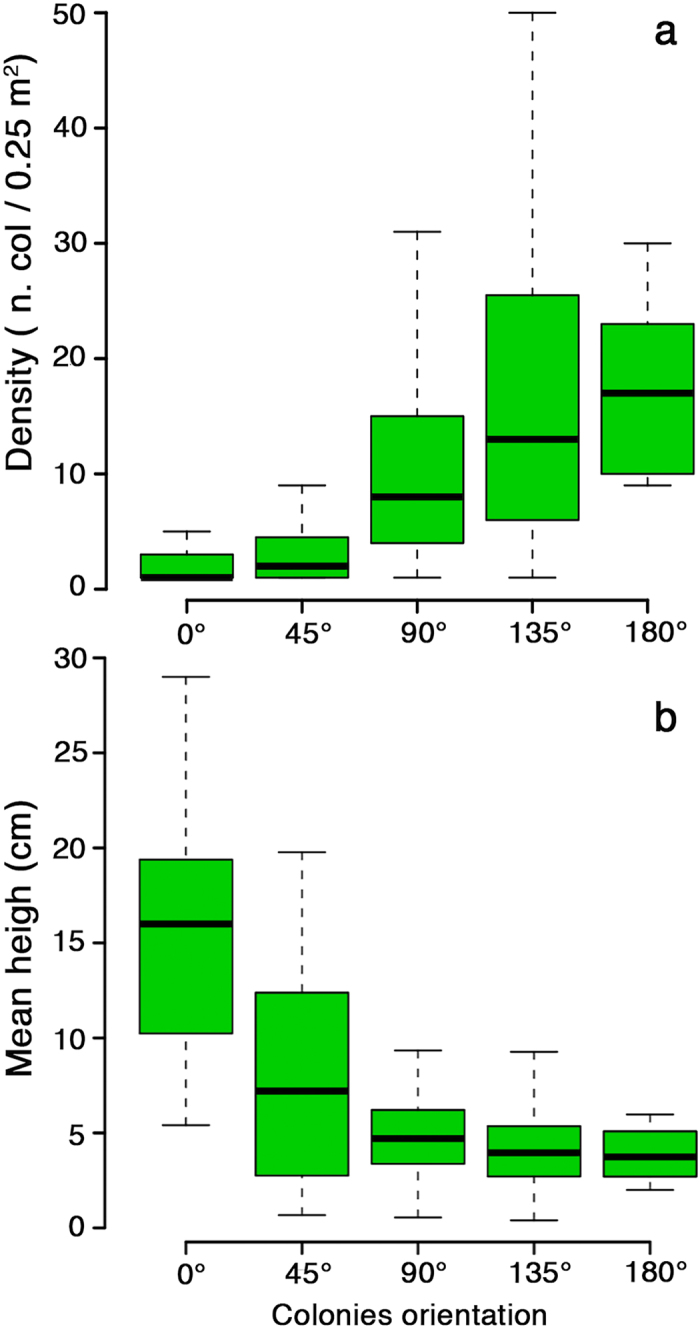
Box plot of: (**a**) density, (**b**) mean height, in relation to colony orientation (0°, 45°, 90°, 135° and 180°).

Similarly, at local scale (*i.e*., tens of meters), the highest population density was found on overhanging substrates, decreasing significantly when the substrate faced upwards (Mann-Whitney, *U* = 1.698^4^, *P* < 0.0001; Fig. 4a). On the contrary, the mean colony height decreased significantly from upward facing to overhanging substrates (Mann-Whitney, *U* = 9,138.0, *P* < 0.0001; Fig. 4b).

**Figure 5:**
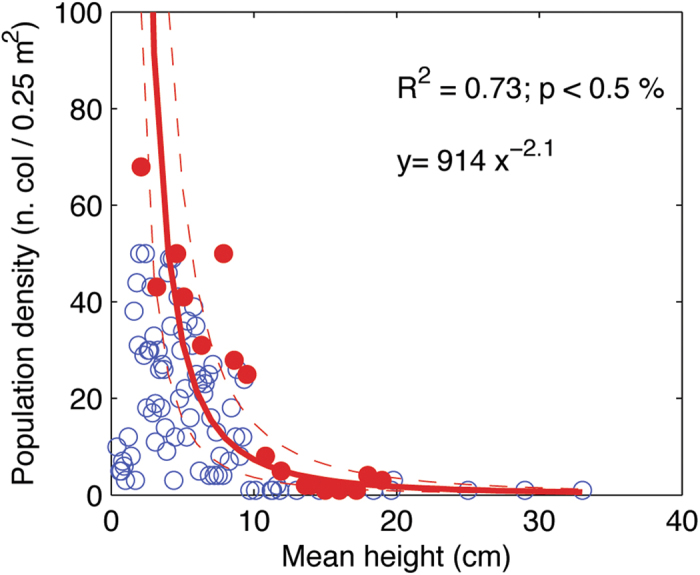
Relationship between density and height (black dots; n = 319). The red dots and line represent the envelope curve and its regression.

In fact, the highest densities are observed only in populations dominated by small colonies, whereas largest and highly branched colonies can be observed only in patches with a density lower than 10 colonies 0.25 m^−2^ (Fig. 5, Supplementary Figure S1). Between these two extremes, colonies of intermediate height and branching pattern can be found at intermediate population densities. For each mean colony height or branching class, population density is variable, but maximum values are inversely related to mean colony height and branching through a negative power curve (*R*^*2*^ = 0.73, *P* < 0.005 for colony height; *R*^*2*^ = 0.62, *P* < 0.005 for branching). Moreover, colony height resulted proportional to -0.48 power of the population density.

Given the small proportion of population density variance explained by depth, the relationship with other local factors that displayed potential co-variation (local slope, sediment presence) on *C. rubrum* presence and density was investigated in the two environments (*i.e*., PIe and VWe) using a General Linear Model. The presence of *C. rubrum* colonies (*P* < 0.001) was negatively related to sediment presence, while no relationship was found between population density and sediment (Supplementary Table S3). In contrast, *C. rubrum* presence was not related to the local slope of the substrate (Supplementary Table S3), but population density was positively related with local slope in both environments (the greater the slope, the larger the population density), showing a stronger relationship in PIe. Water depth explained significant but small proportion of either colonies presence or density variations in both conditions (Supplementary Table S3). No residual pattern was observed for both models.

## Discussion

Our result provide evidence of a size/density relationship within *C. rubrum* populations, typical of erect organisms such as trees and corals^29^,^30^,^35^. A similar relationship has been found for other Mediterranean benthic suspension feeders of the photic zone (*e.g*., *Paramuricea clavata* and *Eudendrium racemosum*)^30^,^36^. The presence of this relationship suggests that self-thinning processes are regulating population density and structure. These processes include recruitment limitation and the differential survival of size classes, which result in the adjustment of population density to the growth of organisms under space limitation. The presence of a self-thinning mechanism was inferred by Yoda *et al.*^35^ in several trees species from the observation that mean biomass (hence volume, given a constant body density) is proportional to -3/2 power of population density in plant populations at limiting density. In the present study, colony height resulted proportional to -0.48 power of population density, a value close to the -1/2 power fit expected for self-thinned populations of organisms with a cubic shape (volume being height to the power of three). However, for organisms with different shapes, the exponent of the population density/individual height power fit might be different^29^.

Existing demographic models for red coral populations, which incorporate a self-thinning process through recruitment limitation by space, foresee an alternation between two structures in natural populations: small and densely packed (the so called ‘grass-plain like’ structure) *versus* large and sparsely distributed colonies (the ‘forest like’ structure)^13^,^37^. In species for which population structure is driven by self-thinning processes, high densities are characteristic of immature or disturbed populations, whereas mature or stable populations display low densities of large colonies (but low densities does not imply stable or mature populations). As a consequence, the finding that only high-density populations can be observed in shallow environments while low density mature populations can be found in deep ones is consistent with a strong depth cut off at 50 m in the selective harvesting pressure on this species, along with environmental disturbances^23^,^25^.

These observations have several implications for *C. rubrum* conservation and management practice.

The absence of baseline information on the pre-fishing density of deep-dwelling red coral populations does not allow us to define observed mature populations as “pristine”^14^,^38^. However, our results suggest that the observed demography of deep dwelling populations might represent a snapshot of the structure of past shallow populations, before the latter were modified by intensive harvesting pressure.

An efficient and long lasting protection should therefore result in a tendency of shallow populations to resemble to deep-dwelling ones, with both immature (dense and small) and stable (sparse and large) populations. This hypothesis is supported by data from literature showing that the oldest MPAs (more than 30 years of strict enforcement) host populations with lower density and larger size than those observed in unprotected populations dwelling at the same shallow depth^39^. Secondly, monitoring of red coral population recovery in MPAs currently includes mean colony size, population density and population size structure assessment in a few patches of the species spatial distribution as index of protection efficiency^39^. According to our results, population density is a counterintuitive and unreliable index. It is counterintuitive because a decay in population density may not be perceived as a positive indicator of protection efficiency by general public although in self-thinned population such a decay is an indicator of population stability. It is unreliable because the wide variability in the density of young populations is related to a complex array of environmental filters on the species life cycle (settlement and post settlement survival) and not only to protection efficiency. Neither mean colony size, on the long term, may prove to be a good index of protection efficiency as it is expected to exhibit periodic variations at the species life expectancy timescale^13^. An efficient protection, in fact, should imply multi-generational species persistence with spatial distribution extending to all suitable habitats (including harvested sites), resulting in the coexistence of dense patches of small colonies (recent colonization) and sparse patches of large ones (oldest colonization). Thus, we suggest that MPAs monitoring should include species spatial distribution at the habitat scale as an indicator of recovery of red coral stocks.

For sessile species, colonization of new substrates depends on their ability to disperse, settle and recruit. On the basis of genetic studies showing fragmentation in shallow and deep populations^4^,^19^,^40^, the role of larval dispersal in the recovery of *C. rubrum* populations have been neglected. Instead, restoration methods based on transplants have been proposed to increase species coverage and accelerate the recovery process^41^. In this view, transplantation efficiency could be improved by choosing the density of transplanted patches on the basis of self-thinning results, *i.e.*, avoiding high densities of colonies that would anyway become sparser through intra-specific competition. Notwithstanding the interest of transplant experiment to foster populations’ recovery for such a slow growing species, its efficiency at large scale is questionable compared to larval dispersal. Although genetic studies found a deficit of effective connectivity between populations^4^,^19^,^43^ compared to genetic drift, *Corallium rubru*m larval traits do not limit the potential dispersal over several km^42^. Nevertheless, the high genetic structuration of *C. rubrum* populations highlighted on metrics integrating gene flow over evolutionary time scale, do not exclude the existence of sporadic migration events between populations^44^. Thus, it cannot be excluded that larval dispersal has a role in determining the distribution of deep red coral populations in the highly fragmented seascape characterised by small rocky outcrops separated by few miles of soft bottoms.

However, other environmental constraints could limit settlement and recruitment of *C. rubrum* in empty substrates. The presence of large colonies in the low productivity deep plain environment (compared to shallow environment)^21^ suggests that red coral distribution is not limited by low temperature, high hydrostatic pressure or low organic matter supply. Thus, depth should not be used to define the habitat of this species. However substrate suitability can be a limiting factor, acting as bottleneck for larval settlement along the complex life cycle of marine benthic invertebrates^45^. Although the significant structuring effect of geomorphological factors on *C. rubrum* populations (*i.e*., immature and mature populations were observed mainly in canyons and deep plains respectively), red coral settlement was not limited by substrate orientation. These differences in population structure should probably be related to the frequency of disturbance that is expected to be higher in canyons than in deep plains (dense water cascading and turbidity flows)^46^, and/or to a low mechanical stability of large colonies on vertical or overhanging substrates. In contrast, the fact that the largest colonies (>25 cm in height, *i.e*., those actually relevant for economic purposes) were only found in horizontal substrates with low sediment, suggests that sediment limits the presence of *C. rubrum*, probably affecting the early stages of the life cycle (adherence deficiency or asphyxia, filtration clogging^47^). These findings are in line with observations recently reported from the Tyrrhenian sea^18^. To better define the red coral physical habitat, the causal relationship either between flow intensity and suspended particulate matter concentration and colony survival, or between sedimentation and larval settlement, should be investigated.

Finally, on the basis of the results obtained by *non-invasive* surveys along different geomorphologies of the continental margin (*i.e*., rocky outcrops and canyons), we were able to challenge the paradigm of “the deeper the habitat, the larger and sparser the colonies” based on previous deep surveys, to provide evidence for self-thinned *C. rubrum* populations and to redefine habitat constraints for this species. Our results supply a new theoretical framework for the assessment of the efficiency of conservation and management strategies *of C. rubrum* populations.

## Methods

### Study area

The investigated area is located in the southern waters of Sardinia, in the central-western region of the Mediterranean Sea, between 80 and 170 m depth (Fig. 1). The southeast and southwestern coasts of Sardinia are characterised by different seafloor morphologies. The western side shows a vast shelf area that is characterised by numerous volcanic outcrops, whereas a considerably smaller shelf area (2 km in extent) with several canyon heads occurs along the south-eastern shelf margin (Fig. 1)^48^,^49^. A total of 12 sites were investigated, each corresponding to a ROV transect (Fig. 1; Table S3); the bathymetric range extended from 80 m in the proximal area to 170 m in the distal area of the continental shelf.

### Data acquisition

Biological data on *C. rubrum* were collected during the autumn 2011 ROV campaign carried out on board the I.S.P.R.A r/v “Astrea”. The ROV “Pollux III” was equipped with a digital camera (Nikon D80, 10 megapixel), a strobe (Nikon SB 400), a high-definition video camera (Sony HDR-HC7) and three laser beams providing a 10-cm scale for the measurement of frames, sampling units and colony size. The ROV was equipped with a depth sensor, a compass and an underwater acoustic tracking positioning system (Tracklink 1500 MA, LinkQuest Inc.). Transects could not be linear as the survey focused on the target species *C. rubrum*, which is distributed on hard bottom patches which include steep walls, caves and boulders^50^.

More than 18 hours of ROV footage was obtained, conducted at a mean speed of 0.35 knots. The precise length of each track (time from reaching the bottom to leaving the bottom) was obtained through the ROV Track-link system.

### Image analysis

Frames were randomly extracted from the high-resolution ROV footage using the software “DVDVIDEOSOFT” and classified according to the quality, discarding overlaying and low-visible frames, keeping the usable ones (50 randomly chosen usable frames per each site). The frame analysis was performed with CPCe (Coral Point Count with Excel extensions) Software^51^, using 0.5 × 0.5 m squares as a randomly positioned sampling unit within each frame. The use of 0.25 m^2^ sampling units was a worthwhile working solution, as frames were not always larger than one square meter, because of the contemporary necessity of “close-viewing” frames to facilitate and improve measurement precision.

In order to investigate how habitat constrains species occurrence (presence) and population structure, kilometric scale (ROV transect) and metric scale (sampling unit of 0.25 m^2^) habitat descriptors were considered. At the kilometric scale, mean slope for each site (*i.e.*, ROV transect) was calculated by averaging slope values estimated from 5 × 5 meters cells of multi-beam bathymetric data along the ROV transect. Each site was subsequently attributed to one of two geomorphological environments defined as: canyon or “vertical wall” environment (“VWe”) with slope larger than 40° and rocky outcrops such as shoals or pinnacles (“PIe”) with slope lower than 40°.

At the metric scale, overall, 600 sampling units were processed, estimating the probability of occurrence (percentage of quadrats in which red coral was found). In addition, the following parameters were retrieved: i) density, estimated as number of colonies per 0.25 m^2^ (0.5 × 0.5 m quadrat); ii) colony height (from the basis to the furthest tip^7^,^17^); iii) local slope of the substrate (expressed as vertical or horizontal wall); iv) orientation of colonies, with respect to the substrate: 0° (vertically oriented colonies), 45°, 90°, 135°, 180° (colonies in overhanging position); v) branching pattern^52^ and vi) silt presence or absence.

### Correlation with depth

In order to test the influence of depth on red coral population density, linear regressions were tested between mean population density and depth, spanning different bathymetric range. Linear regression were performed among (1) data of the present study (12 sites, 80–170 m depth range), (2) all published data from the deep environment (<50 m depth), including the present study (38 sites overall, 50–170 m depth range) and (3) all published data over the entire bathymetric range, including present study (53 sites, 20–170 m depth range). The source of published datasets from shallow and deep environment included in the linear regressions are detailed in Supplementary Table S2.

### Colony size distribution.

The size distribution of the two investigated habitats was analysed with the software XLSTAT (‘data description’ function), using skewness and kurtosis as parameters. Skewness is a measure of the symmetry of a distribution, using its mean; if significant, the distribution is asymmetric. Positive skewness indicates a population mainly composed by small size colonies, while negative skewness indicates the dominance of large size classes^30^,^53^.

### Statistical analyses

Tests were performed on the whole dataset and for the two environmental categories (PIe and VWe), using the software XLSTAT (function ‘normality tests’), to perform both the Shapiro-Wilk and Anderson-Darling tests. Both tests were significant, for both analyses (*P* < 0.0001). Following verification of a non-normal distribution, a uni-variate non-parametric analysis of variance (Mann-Whitney procedure), using the software PAST 2.17^54^, was performed to test for differences in *C. rubrum* density (square rooted data), between the two types of environments: VWe vs. PIe (total n = 600).

The Poisson distribution was verified through XLSTAT software, function ‘distribution fitting’ (n = 600; *P* < 0.0001). Given the large number of zeros in the entire dataset (n = 304; 50.7%), a General Linear Model (GLM) with Zero Altered Poisson distribution (ZAP; R software Version 2.15.1^55^, ‘hurdle’ function) was used to describe how coral density varies in relation to different environmental factors and covariates. The Poisson distribution was preferred to the Negative Binomial because of the absence of extra overdispersion in the non-zero data^55^, whereas the total overdispersion in PIe and VWe was 3.44 and 2.82, respectively, which was considered an acceptable value with respect to dataset features. The response variable was the value of density (number of colonies in each sampling unit, 0.25 m^2^), in relation to the following predictive variables: presence or absence of silt, local slope (vertical/horizontal), and depth as a covariate. The sampling design included exclusively rocky bottoms, which are all suitable substrates for *C. rubrum* settlement avoiding the possibility of false zeros. The Vuong test (command ‘vuong’) showed that a hurdle model fitted the dataset significantly better than a standard GLM with a Poisson distribution (*P* < 0.0001), and no residual pattern was observed. In addition, the ‘hurdle’ function was preferred to the ‘zeroinfl’, as it considers all zeros as ‘true zeros’ and gave us the opportunity to evaluate the explanatory power of variables on ‘non-zeros’ (*i.e.*, population density, truncated Poisson model) and ‘zeros’ (binomial model)^55^,^56^ separately. The model was applied to the two environments (VWe and PIe). The best fit was given by the lowest Akaike information criterion (AIC).

### Self-thinning evaluation

The occurrence of self-thinning has been evidenced by population density decay when the mean colony grows^36^ due to intra-specific competition for a limited resource (hereby space). However, such relationship is expected only in populations at the maximum saturating density. In the general case of natural populations, population density might be lower than the maximum saturating density after disturbances or due to limited reproductive input. In order to detect self-thinning in a dataset potentially including disturbed and undisturbed populations of red coral, the existence of an inverse relationship between the mean space occupied by a colony and population density was examined considering only the largest observed density; in other words, those that are possibly approaching the saturating density.

Colony height and branching were used as proxies of the mean space occupied by a colony. Eighteen size classes of width 1cm were defined for height ranging from 2 to 21 cm and 14 classes of width 1 were defined for branching ranging from 1 to 14. Maximum population density was detected within each height or branching class and a power curve was fitted between population density maximum values and corresponding branching or height (Fig. 5, Supplementary Figure 1). Data were log-log transformed to linearize the power relationship and a linear regression was tested and used to derive power curve fit parameters. The power fit excluded height smaller than 2 cm and branching smaller than 1 due to uncertainties in detection of small colonies that tend to underestimate population density. Branching values larger than 14 and height values larger than 21 cm, which represent a small proportion of the dataset (less than 6%), were excluded to avoid biasing the power fit by over-representation of largest values. Self-thinning was demonstrated by a significant negative slope of the linear regression and the slope was the exponent of power fit.

## Additional Information

**How to cite this article**: Cau, A. *et al.* Habitat constraints and self-thinning shape Mediterranean red coral deep population structure: implications for conservation practice. *Sci. Rep.* **6**, 23322; doi: 10.1038/srep23322 (2016).

## Supplementary Material

Supplementary Information

## References

[b1] Gili J. M. & Coma R. Benthic suspension feeders: their paramount role in littoral marine food webs. Trends Ecol. Evol. 13, 316–21 (1998). 21238320 10.1016/s0169-5347(98)01365-2

[b2] Cerrano C. *et al.* Gold coral (Savalia savaglia) and gorgonian forests enhance benthic biodiversity and ecosystem functioning in the mesophotic zone. Biodivers. Conserv. 19, 153–167 (2010).

[b3] Tsounis G., Grigg R. & Gili J. The exploitation and conservation of precious corals. Oceanogr. Mar. Biol. An Annu. Rev. 48, 161–212 (2010).

[b4] Cannas R. *et al.* Genetic monitoring of deep-water exploited banks of the precious Sardinia coral Corallium rubrum (L. 1758): useful data for a sustainable management. Aquat. Conserv. Mar. Freshw. Ecosyst. (2014). 10.1002/aqc.2522

[b5] Taviani M., Freiwald A., Beuck L., Angeletti L. & Remia A. The deepest known occurrence of the precious red coral Corallium rubrum (L. 1758) in the Mediterranean Sea. In: Bussoletti E., Cottingham D., Bruckner A., Roberts G., Sandulli R. (Eds), Proceedings of the International Workshop on Red Coral Science, Management, Trade: Lessons from the Mediterranean. NOOA Technical Memorandum, Silver Spring, MA, CRCP-13, 87–93 (2010).

[b6] Rossi S. *et al.* Survey of deep-dwelling red coral (Corallium rubrum) populations at Cap de Creus (NW Mediterranean). Mar. Biol. 154, 533–545 (2008).

[b7] Follesa M. C. *et al.* Deep-water red coral from the island of Sardinia (north-western Mediterranean): a local example of sustainable management. Mar. Freshw. Res. 64, 706–715 (2013).

[b8] Galasso M. Pesca del Corallium rubrum in Sardegna nell’antichità: materiali e strumenti. In: L’Africa Romana: lo spazio merittimo del Mediterraneo occidentale. Atti del XIV Convegno di Studio, 7–10 December 2000, Sassari, Italy. Rome: Khanoussi *et al.* (eds) Carocci editore, Vol. 1, 1159–1200.

[b9] Santangelo G., Carletti E., Maggi E. & Bramanti L. Reproduction and population sexual structure of the overexploited Mediterranean red coral Corallium rubrum. Mar. Ecol. Prog. Ser. 248, 99–108 (2003).

[b10] Priori C. *et al.* Demography of deep-dwelling red coral populations: Age and reproductive structure of a highly valued marine species. Estuar. Coast. Shelf Sci. 118, 43–49 (2013).

[b11] Santangelo G. & Abbiati M. Red coral: conservation and management of an over-exploited Mediterranean species. Aquat. Conserv. Mar. Freshw. Ecosyst. 259, 253–259 (2001).

[b12] Bramanti L. *et al.* Demographic parameters of two populations of red coral (Corallium rubrum L. 1758) in the North Western Mediterranean. Mar. Biol. (2014). 10.1007/s00227-013-2383-5

[b13] Bramanti L., Iannelli M. & Santangelo G. Mathematical modelling for conservation and management of gorgonians corals: youngs and olds, could they coexist? Ecol. Modell. 220, 2851–2856 (2009).

[b14] Tsounis G., Rossi S., Bramanti L. & Santangelo G. Management hurdles for sustainable harvesting of Corallium rubrum. Mar. Policy 39, 361–364 (2013).

[b15] Cau A., Cannas R., Sacco F. & Follesa M. C. Adaptive management plan for red coral (*Corallium rubrum*) in the GFCM competence area – First part – background information. (2014) FAO, Rome. Document GFCM_SAC15_2013_Inf.22.pdf.

[b16] Gori A. *et al.* Spatial distribution patterns of the gorgonians Eunicella singularis; Paramuricea clavata ;Leptogorgia sarmentosa (Cape of Creus, Northwestern Mediterranean Sea). Mar. Biol. 158, 143–158 (2011).

[b17] Cau A. *et al.* Preliminary data on habitat characterization relevance for red coral conservation and management. Ital. J. Geosci. 134, 60–68 (2015).

[b18] Angiolillo M. *et al.* Distribution and population structure of deep-dwelling red coral in the Northwest Mediterranean. Mar. Ecol. (2015). 10.1111/maec.12274

[b19] Cannas R. *et al.* New insights into connectivity patterns of mesophotic red coral (Corallium rubrum) populations. Hydrobiologia 759, 63–73 (2015).

[b20] Santangelo G., Bramanti L. & Iannelli M. Population dynamics and conservation biology of the over-exploited Mediterranean red coral. J. Theor. Biol. 244, 416–23 (2007). 17064734 10.1016/j.jtbi.2006.08.027

[b21] Segar D. A. In: Introduction to ocean sciences, 3^rd^ edn, 1^st^ electr. edn, version 3.2. Douglas A. segar (edr), Ch. 14, 347-395. (2012).

[b22] Garrabou J. & Harmelin J. G. A 20-year study on life-history traits of a harvested long-lived temperate coral in the NW Mediterranean: insights into conservation and management needs. J. Anim. Ecol. 71, 966–978 (2002).

[b23] Cerrano C. *et al.* A catastrophic mass-mortality episode of gorgonians and other organisms in the Ligurian Sea (North- western Mediterranean), summer 1999. Ecol. Lett. 3, 284–293 (2000).

[b24] Bramanti L. *et al.* Detrimental effects of ocean acidification on the economically important Mediterranean red coral (Corallium rubrum). Glob. Chang. Biol. 19, 1897–1908 (2013). 23505003 10.1111/gcb.12171

[b25] Cerrano C. *et al.* Red coral extinction risk enhanced by ocean acidification. Sci. Rep. 3, 1457 (2013). 23492780 10.1038/srep01457 PMC3597996

[b26] Torrents O., Tambutté E., Caminiti N. & Garrabou J. Upper thermal thresholds of shallow vs. deep populations of the precious Mediterranean red coral Corallium rubrum (L.): Assessing the potential effects of warming in the NW Mediterranean. J. Exp. Mar. Bio. Ecol. 357, 7–19 (2008).

[b27] Rossi S. The destruction of the ‘animal forests’ in the oceans: Towards an over-simplification of the benthic ecosystems. Ocean Coast. Manag. 84, 77–85 (2013).

[b28] Santangelo G. *et al.* Patterns of variation in recruitment and post-recruitment processes of the Mediterranean precious gorgonian coral Corallium rubrum. J. Exp. Mar. Bio. Ecol. 411, 7–13 (2012).

[b29] Miyanishi K., Hoy a R. & Cavers P. B. A generalized law of self-thinning in plant populations (self-thinning in plant populations). J. Theor. Biol. 78, 439–442 (1979). 513791 10.1016/0022-5193(79)90342-4

[b30] Linares C., Coma R., Garrabou J., Diaz D. & Zabala M. Size distribution, density and disturbance in two Mediterranean gorgonians: Paramuricea clavata and Eunicella singularis. J. Appl. Ecol. 45, 688–699 (2008).

[b31] Li X. Competition-density effect in plant populations. J. For. Res. 13, 48–50 (2002).

[b32] Edinger E. N. *et al.* Geological features supporting deep-sea coral habitat in Atlantic Canada. Cont. Shelf Res. 31, S69–S84 (2011). 10.1016/j.csr.2010.07.004

[b33] Cau A. *et al.* Deepwater corals biodiversity along roche du large ecosystems with different habitat complexity along the south Sardinia continental margin (CW Mediterranean Sea). Mar. Biol. 162, 1865–1878 (2015). 10.1007/s00227-015-2718-5

[b34] Wilson M. F. J., O’Connell B., Brown C., Guinan J. C. & Grehan A. J. Multiscale Terrain Analysis of Multibeam Bathymetry Data for Habitat Mapping on the Continental Slope. Mar. Geod. 30, (2007). 10.1080/01490410701295962

[b35] Yoda K., Kira T., Ogawa H. & Hozumi K. Self-thinning in over- crowded pure stands under cultivated and natural conditions. Intra-specific competition among higher plants. J. Biol. 14, 107–129 (1963).

[b36] Rossi S., Bramanti L., Broglio E. & Gili J. M. Trophic impact of long-lived species indicated by population dynamics in the short-lived hydrozoan Eudendrium racemosum. Mar. Ecol. Prog. Ser. 467, 97–111 (2012).

[b37] Bavestrello G., Bo M., Bertolino M., Betti F. & Cattaneo-Vietti R. Long-term comparison of structure and dynamics of the red coral metapopulation of the Portofino promontory (Ligurian Sea): a case-study for a Marine Protected Area in the Mediterranean Sea. Mar. Ecol. (2014). 10.1111/maec.12235

[b38] Bavestrello G., Bo M., Canese S., Sandulli R. & Cattaneo-Vietti R. The red coral populations of the gulfs of Naples and Salerno: human impact and deep mass mortalities. Ital. J. Zool. 1–12 (2014). 10.1080/11250003.2014.950349

[b39] Linares C. *et al.* Marine Protected Areas and the conservation of long-lived marine invertebrates: the Mediterranean red coral. Mar. Ecol. Prog. Ser. 402, 69–79 (2010).

[b40] Costantini F. *et al.* Low connectivity and declining genetic variability along a depth gradient in Corallium rubrum populations. Coral Reefs 30, 991–1003 (2011).

[b41] Bramanti L., Rossi S., Tsounis G., Gili J. M. & Santangelo G. Settlement and early survival of red coral on artificial substrates in different geographic areas: some clues for demography and restoration. Hydrobiologia 580, 219–224 (2007).

[b42] Martínez-Quintana A., Bramanti L., Viladrich N., Rossi S. & Guizien K. Quantification of larval traits driving connectivity: the case of Corallium rubrum (L. 1758). Mar. Biol. 162, 309–318 (2014). 10.1007/s00227-014-2599-z

[b43] Costantini F., Carlesi L. & Abbiati M. Quantifying spatial genetic structuring in mesophotic populations of the precious coral Corallium rubrum. PLoS One 8, e61546 (2013). 23646109 10.1371/journal.pone.0061546 PMC3640028

[b44] Padron M. & Guizien K. Modelling the effect of demographic traits and connectivity on the genetic structuration of marine metapopulations of sedentary benthic invertebrates. ICES J. Mar. Sci. 10.1093/icesjms/fsv158.

[b45] Scheltema R. On dispersal and planktonic larvae of benthic intvertebrates: an eclectic overview and summary of problems. Bull. Mar. Sci. 39, 290–322 (1986).

[b46] Canals M. *et al.* Flushing submarine canyons. Nature 444, 354–7 (2006). 17108962 10.1038/nature05271

[b47] Perez K., Rodgers K. S., Jokiel P. L., Lager C. V. & Lager D. J. Effects of terrigenous sediment on settlement and survival of the reef coral Pocillopora damicornis. PeerJ 2, e387 (2014). 24883248 10.7717/peerj.387 PMC4034646

[b48] Sulli A. Structural framework and crustal characteristics of the Sardinia Channel Alpine transect in the central Mediterranean. Tectonophysics 324, 321–336 (2000).

[b49] Mascle G. H. *et al.* Evolution of the Sardinia Channel (Western Mediterranean): New constraints from a diving survey on Cornacya seamount off SE Sardinia. Mar. Geol. 179, 179–202 (2001).

[b50] Cannas R. *et al.* The red coral resource in Sardinian seas : a multidisciplinary survey on Corallium rubrum populations. Stud. Trentini di Sci. Nat. 89, 9–18 (2011).

[b51] Kohler K. E. & Gill S. M. Coral Point Count with Excel extensions (CPCe): A Visual Basic program for the determination of coral and substrate coverage using random point count methodology. Comput. Geosci. 32, 1259–1269 (2006).

[b52] Brazeau D. A. & Lasker H. R. Inter- and intraspecific variation in gorgonian colony morphology: quantifying branching patterns in arborescent animals. Coral Reefs 7, 139–143 (1988).

[b53] Edmunds P. J. *et al.* Evaluating the causal basis of ecological success within the scleractinia: an integral projection model approach. Mar. Biol. 161, 2719–2734 (2014).

[b54] Hammer H., Harper D. A. & Ryan P. D. PAST: Paleontological statistics software package for education and data analysis. Palaeontol. Electron. 4, 1–9 (2001).

[b55] Zuur A., Ieno E. N., Walker N., Saveliev A. A. & Smith G. M. Mixed effects models and extensions in ecology with R. (Springer, 2009).

[b56] Jackman S. Bayesian Analysis for the Social Sciences. Wiley Ser. Probab. Stat. (Wiley, 2009).

