# Supplementary material for: Habitat constraints and self-thinning shape Mediterranean red coral deep population structure: implications for conservation practice
Source: Sci Rep. 2016 Mar 18;6:23322. doi: 10.1038/srep23322 (PMC4796916; doi:10.1038/srep23322)
Supplement: Supplementary Information [file srep23322-s1.pdf]

Habitat constraints and self-thinning shape Mediterranean red coral deep population structure: implications for conservation practice

Alessandro Cau[1*], Lorenzo Bramanti[2], Rita Cannas[1], Maria Cristina Follesa[1], Michela Angiolillo[3], Simonepietro Canese[3], Marzia Bo[4], Danila Cuccu[1] & Katell Guizien[2]

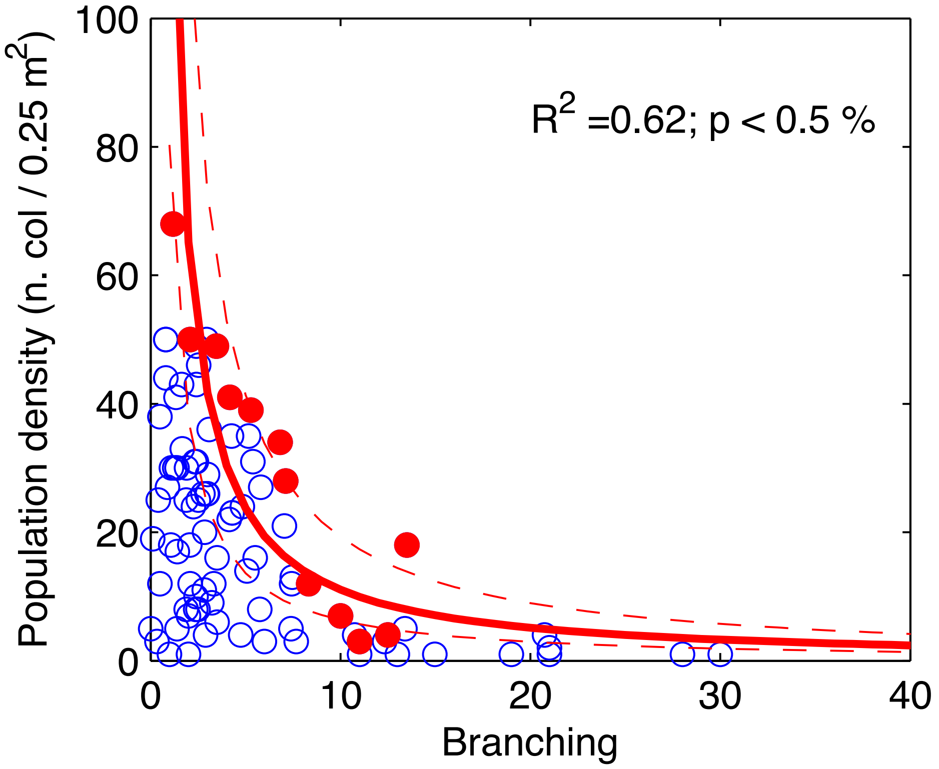

Figure S1: Relationship between branching (n. of branching/ tot. colonies, in each sampling unit) and density per sampling unit. Red dots constitute the envelope line, built using the maximum value of branching in 12 size classes.

Table S1. Geographical coordinates and depth range of the 12 investigated sites.

Site	Latitude (N)	Longitude (E)	Depth range (m)
VWe_1	39°14'369	8°16'684	80-90
VWe_2	39°03'998	9°29'775	120-130
VWe_3	39°06'719	9°25'970	115-130
VWe_4	39°05'414	9°28'327	120-140
VWe_5	39°25'765	9°47'474	120-130
VWe_6	39°24'832	9°44'994	115-130
Ple_1	38°59'816	8°07'720	155-170
Ple_2	39°14'381	8°16'379	90-100
Ple_3	38°44'425	8°29'025	110-120
Ple_4	39°07'173	8°08'258	125-130
Ple_5	39°09'213	8°10'180	80-85
Ple_6	39°05'349	8°07'227	120-130

Table S2. Sites names, depth, mean density and references of the 53 sites retrieved from available scientific literature.

Sites	Country	Depth	Mean Density	Reference
Corsica_La Revellata	France	40	25.20	Gallmetzer *at al.* 2010
Banyuls	France	24	34.25	Linares *et al.* 2010
Carry	France	24.5	11.75	Linares *et al.* 2010
Scandola	France	20.5	17.50	Linares *et al.* 2010
Masina	Spain	72.5	10.50	Rossi *et al.* 2008
Massa d'oros	Spain	65	11.25	Rossi *et al.* 2008
Cap de Creus	Spain	32.5	74.38	Bramanti *et al.* 2014
Costa Brava	Spain	31.5	31.75	Tsounis *et al.* 2006
Tuscany archipelago	Italy	91	12.50	Priori *et al.* 2013
Ischia	Italy	85	16.12	Priori; PhD Thesis
Elba-Pianosa	Italy	85	12.63	Priori; PhD Thesis
Amalfi	Italy	110	35.56	Priori; PhD Thesis
Alghero	Italy	102.5	1.33	Priori; PhD Thesis
VWe_1	Italy	85	15.10	Present study
VWe_2	Italy	125	8.08	Present study
VWe_3	Italy	122.5	1.90	Present study
VWe_4	Italy	130	19.50	Present study
VWe_5	Italy	125	5.34	Present study
VWe_6	Italy	110	1.80	Present study
PIe_1	Italy	170	9.08	Present study
PIe_2	Italy	95	2.27	Present study
PIe_3	Italy	115	1.12	Present study
PIe_4	Italy	127.5	1.25	Present study
PIe_5	Italy	95	3.70	Present study
PIe_6	Italy	125	0.22	Present study
Calabria_1	Italy	100	1.61	Angiolillo *at al.* 2009
Calabria_2	Italy	105	4.51	Angiolillo *at al.* 2009
Calabria_3	Italy	130	24.14	Angiolillo *at al.* 2009
Capo Carbonara	Italy	115	23.00	Cau *et al.* 2015
San Pietro Island	Italy	85	16.00	Cau *et al.* 2015
Portofino promontory_1	Italy	30	75.00	Bavestrello *et al.* 2009
Portofino promontory_2	Italy	50	22.50	Bavestrello *et al.* 2009
Capo di conca	Italy	55	15.50	Bavestrello *et al.* 2014
Secca del Pampano	Italy	140	1.03	Bavestrello *et al.* 2014
Scoglio D'Ischia	Italy	75	3.45	Bavestrello *et al.* 2014
Punta S.Angelo	Italy	91.5	6.00	Bavestrello *et al.* 2014
Li galli	Italy	100	19.4	Bavestrello *et al.* 2014
Punta Solchiaro	Italy	57	24.00	Bavestrello *et al.* 2014
Punta Pizzato	Italy	55	8.25	Bavestrello *et al.* 2014
Punta S.Angelo_2	Italy	91.5	36.50	Bavestrello *et al.* 2014
Punta del faro	Italy	75	10.25	Bavestrello *et al.* 2014b
Isuela shoal	Italy	50	24.25	Bavestrello *et al.* 2014b
Maledetti shoal	Italy	85	69.50	Bavestrello *et al.* 2014b
Bordighera	Italy	70	8.75	Bavestrello *et al.* 2014b

North Pianosa Is.	Italy	75	12.00	Bavestrello *et al.* 2014b
Sante shoal	Italy	87	5.25	Bavestrello *et al.* 2014b
Montecristo shoal	Italy	60.7	18.00	Bavestrello *et al.* 2014b
Tuna paradise	Italy	88	10.25	Bavestrello *et al.* 2014b
Portofino_Isuela Sud	Italy	32.5	56.75	Bavestrello *et al.* 2014c
Amalfi	Italy	71	8.89	Angiolillo e*t al.* 2015
Elba	Italy	79	12.63	Angiolillo e*t al.* 2015
Ischia	Italy	84	16.11	Angiolillo e*t al.* 2015
Portofino promontory	Italy	35	250	Bramanti et al. 2014

References:

1. Bavestrello, G., Bo, M., Canese, S., Sandulli, R. & Cattaneo-Vietti, R. The red coral populations of the gulfs of Naples and Salerno: human impact and deep mass mortalities. *Ital. J. Zool.* 1–12 (2014). doi:10.1080/11250003.2014.950349

2. Bavestrello, G., Bo, M., Bertolino, M., Betti, F. & Cattaneo-Vietti, R. Long-term comparison of structure and dynamics of the red coral metapopulation of the Portofino Promontory (Ligurian Sea): a case-study for a Marine Protected Area in the Mediterranean Sea. *Mar. Ecol.* (2014). doi:10.1111/maec.12235

3. Priori, C. *et al.* Demography of deep-dwelling red coral populations: Age and reproductive structure of a highly valued marine species. *Estuar. Coast. Shelf Sci.* **118,** 43–49 (2013).

4. Angiolillo, M. *et al.* Distribution and population structure of deep-dwelling red coral in the Northwest Mediterranean. *Mar. Ecol.* (2015). doi:10.1111/maec.12274

5. Cau, A. *et al.* Preliminary data on habitat characterization relevance for red coral conservation and management. *Ital. J. Geosci.* **134,** 60–68 (2015).

6. Follesa, M. C. *et al.* Deep-water red coral from the island of Sardinia (north-western Mediterranean): a local example of sustainable management. *Mar. Freshw. Res.* **64,** 706–715 (2013).

7. Tsounis, G., Rossi, S., Gili, J.-M. & Arntz, W. Population structure of an exploited benthic cnidarian: the case study of red coral (Corallium rubrum L.). *Mar. Biol.* **149,** 1059–1070 (2006).

8. Rossi, S. *et al.* Survey of deep-dwelling red coral (Corallium rubrum) populations at Cap de Creus (NW Mediterranean). *Mar. Biol.* **154,** 533–545 (2008).

9. Gallmetzer, I., Haselmair, A. & Velimirov, B. Slow growth and early sexual maturity: Bane and boon for the red coral Corallium rubrum. *Estuar. Coast. Shelf Sci.* **90,** 1–10 (2010).

10. Bramanti, L. *et al.* Demographic parameters of two populations of red coral (Corallium rubrum L. 1758) in the North Western Mediterranean. *Mar. Biol.* (2014). doi:10.1007/s00227-013-2383-5

11. Linares, C. *et al.* Marine Protected Areas and the conservation of long-lived marine invertebrates: the Mediterranean red coral. *Mar. Ecol. Prog. Ser.* **402,** 69–79 (2010).

12. Angiolillo, M. *et al*. Presence of Corallium rubrum assemblages below 50 m along the Calabrian coasts. In: UNEP-MAP-RAC/SPA, 2009. Proceedings of the 1th symposium on conservation of the coralligenous bio-concretions (Tabarka 16e19 January 2009). Pergent-Martini C., Brichet (eds) RAC/SPA publ. Tunis.

13. Bavestrello, G. *et al*. Remotely Operated Vehicles (ROVs) as powerful tools for the evaluation of the conservation status of deep red coral banks. In: Bouafif C., Langar H., Ouerghi A. (Eds), UNEP/MAP–RAC/SPA, Proceedings of the second Mediterranean Symposium on the conservation of Coralligenous and other Calcareous Bio-Concretions (Portoro z, Slovenia, 29–30 October 2014). Tunis: 31–36.

Table S3: Output of the GLM (ZAP model, function 'hurdle') ran for both environments (i.e. PIe and VWe); (N.S. Not Significant; * P-value<0.05; ** P-value<0.01; *** P-value<0.001).

**Call for "PIe" environment**				
**hurdle(formula = density ~ depth + local slope + accumulated sediment I depth + local slope + accumulated sediment, data)**				
**Count model coefficients (truncated Poisson with log link):**				
	**Estimate**	**Std. Error**	**Z value**	**P Value**
Intercept	0.682	0.150	4.54	***
Depth	0.009	0.001	7.95	***
Local slope (vertical)	0.34	0.074	4.57	***
Accumulated sediment (yes)	-0.28	0.1	-2.80	**
**Zero hurdle model coefficients (binomial with logit link):**				
	**Estimate**	**Std. Error**	**Z value**	**P Value**
Intercept	-3.94	0.77	-5.11	***
Depth	0.039	0.007	5.61	***
Local slope (vertical)	0.11	0.30	0.37	N.S.
Accumulated sediment (yes)	-2.938	0.40	-7.43	***

**Call for "VWe" environment**				
**hurdle(formula = density ~ depth + local slope + accumulated sediment I depth + local slope + accumulated sediment, data)**				
**Count model coefficients (truncated Poisson with log link):**	**Estimate**	**Std. Error**	**Z value**	**P Value**
Intercept	1.656	0.192	8.62	***
Depth	0.008	0.001	3.91	***
Local slope (vertical)	0.295	0.087	3.37	***
Accumulated sediment (yes)	-2.181	0.650	-3.36	***
**Zero hurdle model coefficients (binomial with Logit link):**				
	**Estimate**	**Std. Error**	**Z Value**	**P Value**
Intercept	1.395	1.415	0.98	N.S.
Depth	-0.0026	0.013	-0.38	N.S.
Local slope (vertical)	0.2672	0.512	0.56	N.S.
Accumulated sediment (yes)	-4.388	0.812	-5.4	***
